# Custom-Made Device (CMD) for the Repair of Thoraco-Abdominal Aneurysm (TAA): Mid-Long Term Outcomes from a Single Southeast Asian Centre Experience in Singapore

**DOI:** 10.3390/jcm13206145

**Published:** 2024-10-15

**Authors:** Nick Zhi Peng Ng, Jolyn Hui Qing Pang, Charyl Jia Qi Yap, Victor Tar Toong Chao, Kiang Hiong Tay, Tze Tec Chong

**Affiliations:** 1 Department of Vascular Surgery, Singapore General Hospital, Outram Road, Singapore 169856, Singapore; 2 Department of Cardiothoracic Surgery, National Heart Centre Singapore, 5 Hospital Drive, Singapore 169609, Singapore; 3 Department of Vascular and Interventional Radiology, Singapore General Hospital, Outram Road, Singapore 169856, Singapore; 4 Duke-NUS Graduate Medical School, 8 College Road, Singapore 169857, Singapore

**Keywords:** thoraco-abdominal aneurysms, thoracic endovascular aneurysm repair, custom-made devices, endografts

## Abstract

**Introduction:** Given the high risk of peri-operative morbidity and mortality associated with open repair, endovascular repair for thoraco-abdominal aneurysms is increasingly performed. This study aims to describe mid to long-term results for patients who were treated with COOK Custom-Made Endograft Device at a single Southeast Asian tertiary centre. **Methods:** Mid to long-term results of patients treated from 2012 to 2022 were retrospectively reviewed. Indications for treatment were aortic diameter > 5.5 cm, enlargement > 5 mm in 6 months or high-risk morphology. Clinical, operative, early to late complications and reintervention details were captured. The endpoints were technical success, primary patency and primary assisted patency. **Results:** Electronic medical records of 29 consecutive patients (64.4 ± 1.6 years old; 26/29 males 89.6%) were reviewed. 24/29 (83%) were hypertensive, and 20/29 (69%) were smokers. The mean diameter was 5.5 cm, and the majority were treated for Crawford type IV (19/29, 65.5%). Endograft deployment was 100%. Catheterisation of fenestration was successful in 109/116 (94%). 30-day mortality and morbidity were observed in 12/29 (41%), for which access site complications were most common. No significant haemorrhage or graft explant was recorded. The mean follow-up period was 32.4 months (range 1–108 months). Primary patency was 92.9% (95% CI: 83.8–100.0) at 6 months and decreased to 77.7% (95% CI: 63.4–95.2) at 24 months. Sac shrinkage or stability was noted in 17/29 (58.6%). Re-intervention was performed in 9/29 (31%) for limb occlusion (2/9, 22.2%), renal artery stent occlusion (1/9, 11.1%) and endoleaks (6/9, 66.6%). Assisted patency was maintained at 100% for 12 months before decreasing to 66.7% (95% CI: 37.9–100.0) at 24 months. **Conclusions:** The study reports the first mid-long-term result in this region, though limited by the sample size. Re-intervention at 30% suggests that disease and procedures remain challenging, emphasising the need to assimilate lessons and experience at high-volume centres.

## 1. Introduction

During the last decade, there has been a shift in the treatment modality of aortic aneurysms towards Endovascular Aortic Repair (EVAR), mainly due to the high peri-operative mortality and morbidity associated with open repair [1,2,3].

While standard EVAR to exclude infra-renal aortic aneurysm sac of the favourable neck is increasingly straightforward and commonplace, EVAR for thoraco-abdominal aneurysm (TAA) and hostile neck aneurysms requires additional considerations and strategies to achieve proximal seal while preserving reno-visceral perfusion [4].

Fenestrated endovascular aortic repair (FEVAR) is one such well-established endovascular treatment option. These devices may be “off-the-shelf” or customised. Fenestrations are holes in the dacron or ePTFE mesh of the stent that can be small, large or semicircular/incomplete holes (scallops) and are reinforced with nitinol wire [5,6].

“Off-the-shelf” devices, while more readily available in emergent settings, can be limited when anatomically unsuitable in 20–50% of patients. Specifically, they are less suitable if the branch vessels arise in close proximity to each other and/or in the presence of accessory renal arteries. In some studies, they have been reported to have higher long-term branch complications [6,7,8,9].

Custom-made devices (CMD), as the name suggests, are specifically ordered, individually tailored, designed and manufactured based on measurements from a patient’s pre-operative CT angiography imaging as opposed to the use of combining standard available devices [6,10,11,12].

They take into account not only the dimensions of the aorta, reno-visceral branches and aneurysm but more importantly, the position of these target vessels in relation to each other. This allows for the orientation and spacing of fenestrations and scallops in the fabric to be planned. They are most useful when the aneurysm can be treated in a less time-sensitive and more elective setting, as they take several weeks to make.

Despite the paradigm shift in treatment towards an endovascular approach, open surgery is still often thought of as the gold standard repair. This is because compared to open repair, endovascular techniques have overall higher aortic-related complications, costs, need for long-term surveillance and increased likelihood for reintervention [5]. Often, these drawbacks are accentuated in non-standard EVAR cases [13].

Several short-term and mid-term outcomes have been reported but are largely based on Caucasian cohorts [6,10,11,12]. Given the paucity of reporting of these outcomes in the Southeast Asian and Asian region, the aim of this study is to report a retrospective analysis of consecutive patients and describe the mid to long-term experience from a single tertiary centre in Singapore with the COOK custom-made endograft device from 2012–2022 for TAAs.

## 2. Materials and Methods

### 2.1. Patient Selection

A retrospective analysis of the electronic database and clinical records of all consecutive patients with TAAs treated with CMD at Singapore General Hospital from 2012 to 2022 was performed. Centralised Institutional Review Board approval was obtained (CIRB Ref 2023/2291). A waiver of consent was granted for the clinical audit of our procedural outcomes.

### 2.2. Operation Planning and Procedure

Indication for treatment was a diameter of more than 5.5 cm, rapid sac enlargement of >5 mm in 6 months or a morphology known to be at high risk of rupture, i.e., saccular, eccentric dilatation [14]. All patients had been discussed at a multidisciplinary team meeting attended by interventional radiologists and vascular surgeons.

CMDs were planned on the basis of CT angiography (CTA) measurements taken within 3 months of the procedure. Suitable proximal and distal landing zones, either previous surgical or native aorta of 2 cm, were employed in all cases.

All procedures were performed in a hybrid angio-suite under general anaesthesia. Spinal cerebrospinal fluid (CSF) drainage was employed prophylactically in all cases in this series. Percutaneous common femoral access, with the deployment of 2 ProGlide™ (Abbott Vascular, Santa Clara, CA, USA) suture-mediated closure devices in a pre-close fashion for each side, was used unless a surgical cutdown was needed. Patients were kept under heparinisation with a target-activated clotting time of ≥250 s.

After endograft deployment, renal and visceral arterial catheterisation was achieved from femoral access via simultaneous ‘side to side’ introduction of smaller sheaths into the larger groin sheath and additional left upper extremity access as required.

Catheterisation of these fenestrations into the branches and stenting were thus performed either antegrade or retrograde via upper extremity or femoral access, respectively. Completion of the fenestration was performed with balloon-expandable covered stents, Begraft, or the Begraft plus (Bentley) delivered through an exchange length sheath.

The bridging stents used were either equal in size or up to 1 mm larger than the target diameter and usually additionally flared at the fenestration with a balloon that was 2 mm larger, ensuring adequate sealing within the main graft.

**Figure 1:**
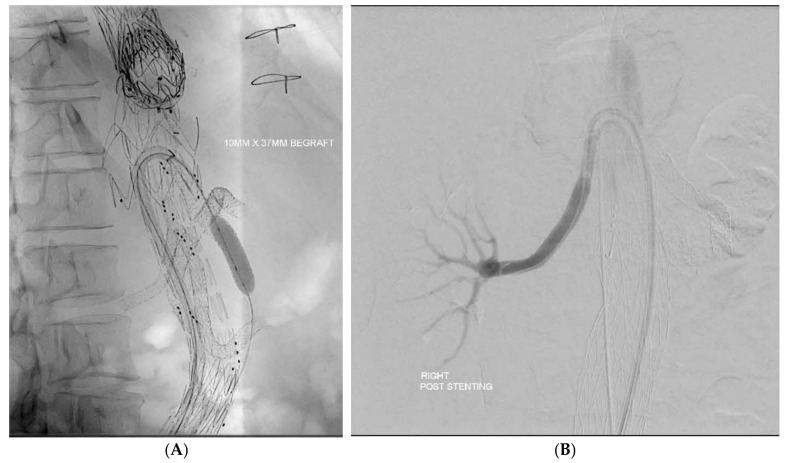
(**A**) The use of APTUS Steerable Sheath for retrograde cannulation is particularly useful in patients with previous TEVAR as well as for (**B**) retrograde cannulation of renal arteries with an acute angle.

The use of the Aptus Endosystems TourGuide steerable sheath (Medtronic, Minneapolis, MN, USA) for retrograde cannulation of visceral is employed, especially when there is known upper limb arterial disease or previous proximal thoracic interventions (Figure 1A,B).

Follow-up was performed with CTA at 30 days, 6 months, 1 year and annually thereafter. In cases in which CTA depicted suspicious findings but did not require immediate re-intervention, stricter imaging surveillance was performed.

### 2.3. Statistical Analysis

We examine the clinical characteristics of the patients, operative technical details such as antegrade versus retrograde catheterisation, use of the steerable sheath, access complications, as well as follow-up results, including limb or branch occlusion, endoleaks and need for reintervention.

Statistical analysis was performed with the SPSS 13.0 statistical package (SPSS Inc., Chicago, IL, USA). Continuous data were described as the mean value ± SD, while non-Gaussian data with median and percentiles. A *p* value < 0.05 was regarded to indicate a statistically significant association. All *p* values were calculated using a two-tailed significance level. A chi-square test was conducted for categorical variables, and a Kruskal-Wallis rank sum test was performed on continuous variables. Logistic regression was performed to identify any predictors for reinterventions, and Kaplan–Meier analysis was performed to measure the fraction of subjects without complications for a certain amount of time after treatment.

### 2.4. Definitions

Endpoints were technical success, complications, primary patency, reinterventions and primary assisted patency.

Technical success was defined as the deployment of the aortic stent graft and modular components, successful catheterisation and placement of bridging stents with flow in all intended target vessels, absence of immediate type I or III endoleaks and patency of all stent graft components at completion angiography.

Primary patency refers to uninterrupted patency without interventions, while primary assisted patency defines the patency of a branch/limb after re-intervention but was reopened successfully.

Reinterventions were defined as procedures designed to treat the underlying aortic disease, such as open conversion, endovascular or intervention for significant endoleaks or branch or limb occlusions.

At follow-up, based on CTA measurements (maximum diameter), aneurysm sac behaviour was classified as reduced (reduction greater than 5 mm), stable and enlarging (increase greater than 5 mm).

## 3. Results

### 3.1. Baseline Demographics and Operative Details

From the time these devices became available in January 2012 till December 2022, 29 consecutive patients (66.7 ± 11.6 years old; 26/29 males (89.7%)) have been treated with the Cook Custom Made Device at Singapore General Hospital. Demographic and clinical characteristics of the study are summarised in Table 1.

A large proportion of patients were hypertensive 24/29 (83%) with smoking history 20/29 (69%). Seven patients had prior coronary artery bypass. 2 had previous TEVAR for thoracic aneurysms, two had open ascending aorta repair, and five patients had open infrarenal aorta repair.

The mean aneurysm diameter was 5.5 cm, and the indication for treatment was a Crawford type IV in 19 patients (65.5%), III in 8 patients (27.6%) and type I in 2 patients (6.9%).

Endograft deployment was achieved in all cases. The custom-made endografts comprised of fenestrations for the coeliac axis, superior mesenteric artery (SMA), both renal arteries, with accessory renal arteries (ARA) in 3 designs. 2 designs did not include fenestrations for the coeliac axis. 2 cases were performed with simultaneous iliac branch device deployment.

Between the cohorts with and without reinterventions, there was a significant difference in the history of aortic valve surgery, open AAA repair and open ascending aorta repair.

Operative details are summarised in Table 2. The mean operative time was 5 h 30 min ± 120 min, ranging from 2.5 to 12 h, and the mean contrast media volume administered during the procedures was 340 ± 93 mL.

### 3.2. Branch Catheterisation

Catheterisation of fenestration/branch was successful in 109/116 (94%) vessels. In 6/27 patients, the coeliac fenestration was not catheterised (33%). In the majority of these, catheterisation was unsuccessful, but as the adequate seal was deemed to have already been achieved, the decision was made not to prolong operative time. For 2 of these patients, a chimney technique was used, and no late endoleak was recorded.

One left renal artery could not be catheterised after multiple attempts, but no significant endoleak was seen at the end of the procedure.

A total of 22/29 (76%) cases had branch cannulation via antegrade proximal access, while the rest were cannulated entirely via retrograde groin access. This was more likely in cases with only three fenestrations and favourable branch angles.

### 3.3. Femoral Access

A total of 27/29 (93%) patients had bilateral percutaneous groin access, while two patients underwent open access in view of vessel calibre and atherosclerotic disease. A conduit was used in one of them.

A total of 4/27 (14%) patients had a failure of closure device necessitating cut down and open repair of the femoral access, for which three were performed for bleeding while 1 for ischemic limb.

A total of 6/29 (20%) common femoral endarterectomies with bovine pericardial patch repair were performed at the access site as they were diseased.

### 3.4. Brachical Access

Percutaneous brachial access complications were not uncommon, with a 4/22 (18%) pseudoaneurysm rate. Eventually, many operators preferred open brachial access to negate this complication altogether.

### 3.5. Adjuncts

Intra-procedural embolisation of branches was performed prior to endograft deployment in seven patients (24%). AVP II plug (Abbott) was most commonly used in internal iliac arteries in two cases, while coils were used for five inferior mesenteric arteries and one accessory renal artery. These were performed as they potentially would have been responsible for an endoleak.

### 3.6. Complications

Peri-procedural (30-day) mortality and morbidity were observed in 12 patients (41%) and are described in Table 3.

Access site complications were most common, with eight returns to the operating theatre for four groin and four brachial access site repairs.

Four perioperative mortalities were recorded, with two patients having post-operative cerebrovascular accidents (CVA), another 2 with cardiac events and one death 11 days from the procedure from pneumonia with NSTEMI.

No significant haemorrhage, aortic perforation or graft explant was recorded.

Three patients experienced post-operative concerns of transient paraplegia, but fortunately, these resolved with CSF drainage.

### 3.7. Follow-Up

The mean follow-up was 32.4 months (range 1–108 months).

Up to the period of follow-up, three additional patients died at 11 months, 16 months and 26 months from non-aortic related pathology, the cause of death being a progression of end-stage renal failure, metastatic prostatic cancer and metastatic gastric cancer, respectively.

CTA follow-up showed sac shrinkage (n = 7) or a stable diameter (n = 10) in 17/29 (58.6%); in the remaining cases (9/29; 31%), sac enlargement was appreciable.

Re-intervention was performed in 9 patients (31%).

Two patients had iliac limb occlusions requiring thrombolysis and stent re-lining, discovered at 6 and 12 months on follow-up, with both presenting as claudication.

**Figure 2:**
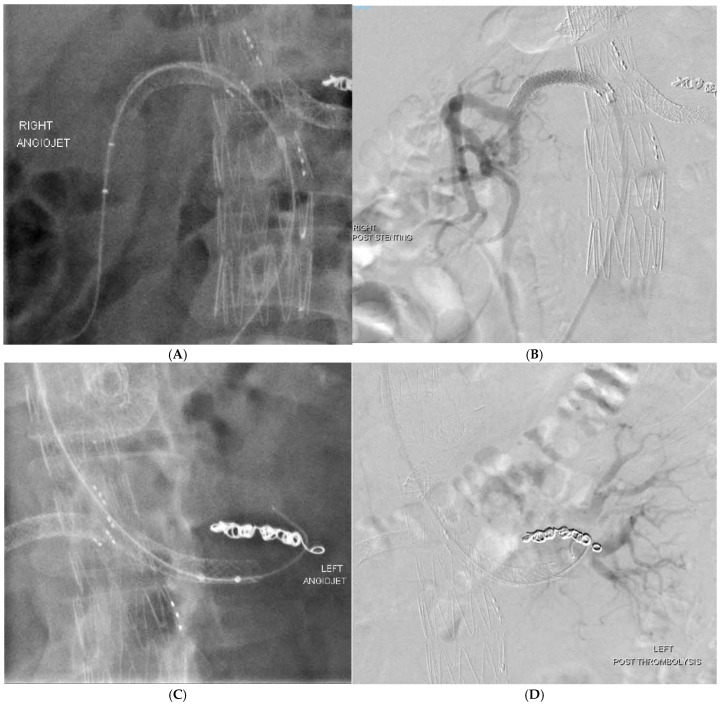
The use of the Angiojet Peripheral Thrombectomy system for thrombolysis of renal branches was demonstrated in 2 cases—(**A**–**D**).

One patient developed acute kidney injury 2 years after the primary procedure secondary to occlusion of bilateral renal artery stents, which resolved with endovascular thrombectomy using the Angiojet Peripheral thrombectomy system (Boston Scientific Corporation, Marlborough, MA, USA) (Figure 2).

Two other patients were found to have unilateral renal stent occlusion on follow-up surveillance CT but were treated conservatively as renal function was not significantly affected.

**Figure 3:**
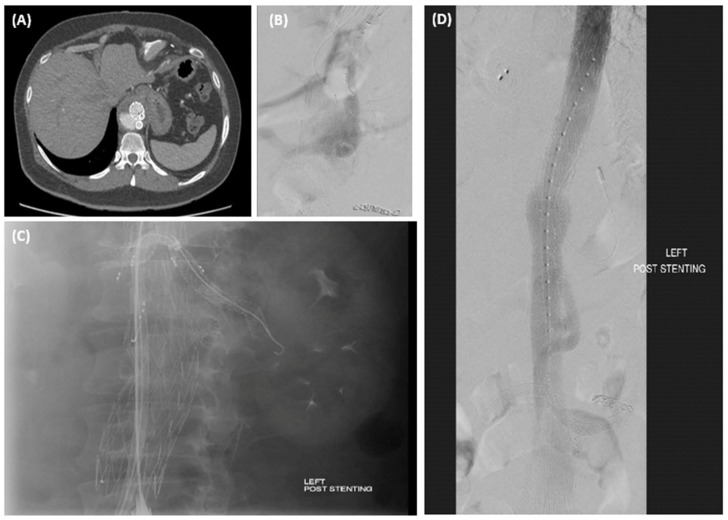
(**A**) Endoleak observed on CT. (**B**) Selective branch cannulation for diagnosis of Type 3 Endoleak. (**C**) Use of steerable sheath for cannulation and stent extension of branches. (**D**) Resolution post-stenting.

Three patients had a branch/stent disconnection, presenting as a type 3 endoleak treated with endovascular relining and stent extension (Figure 3). The steerable sheath has also proved to be useful in these cases for cannulation of the fenestration into the branches.

Two patients with type 1A/B endoleak were treated with main body stent extension and glue injection into the aortic sac space for gutter leaks.

**Figure 4:**
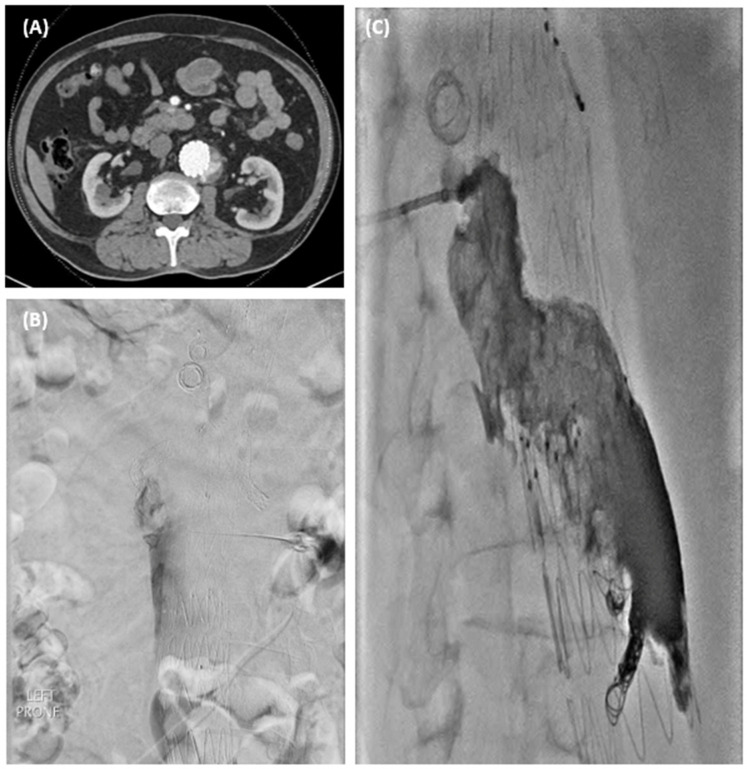
(**A**) CT showing Type 2 endoleak with increasing sac on surveillance. (**B**) Translumbar sac puncture performed under CT guidance with saccogram performed. (**C**) Coil embolisation with Concerto coils of IMA and rest of endoleak cavity filled with glue.

One patient had increasing sac size from type 2 endoleak due to inferior mesenteric artery and underwent trans-lumbar sac puncture under CT guidance followed by coil embolisation with Concerto detachable coils (Medtronic) of IMA and Onyx 18 glue (Medtronic) to sac space as well as the puncture tract (Figure 4).

Univariable logistic regression was performed to identify any significant factors associated with reinterventions. Patients with a history of open aortic repair were identified as one such factor that increases the risk of reintervention (Table 1). As there was only one significant factor identified, multivariable analysis was not performed.

**Figure 5:**
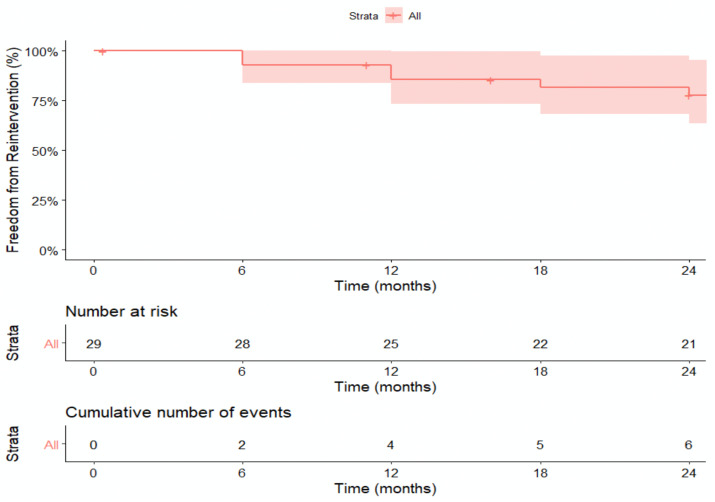
KM Curve for primary patency. Kaplan–Meier estimates for primary patency are 92.9% (95% CI 83.8–100%) at 6 months, 85.4% (95% CI 73.2–99.7%) at 12 months, 81.5% (95% CI 63.4–95.2%) at 24 months.

**Figure 6:**
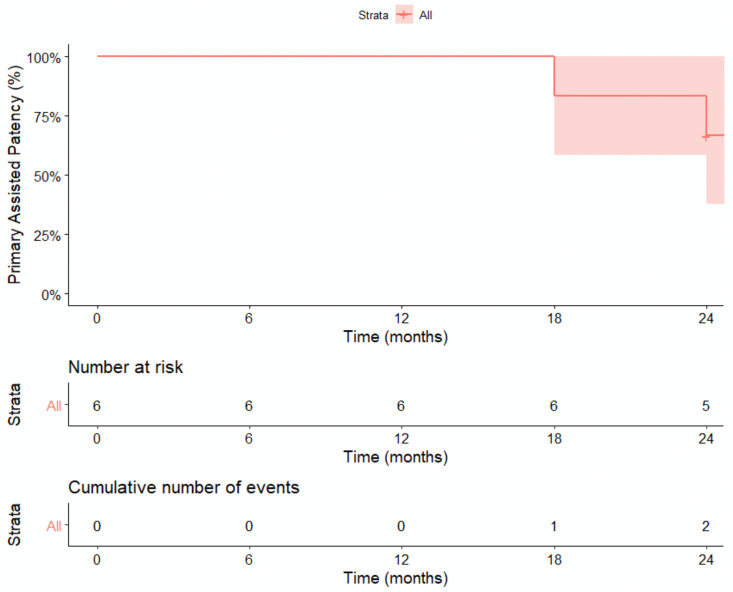
KM Curve for Primary Assisted Patency. Kaplan–Meier estimates for primary assisted patency are 100.0% at 6 and 12 months, 83.3% (95% CI 58.3–100%) at 18 months and 66.7% (95 CI 37.9–100.0%) at 24 months.

Kaplan–Meier curves demonstrate primary patency of 92.9% (95%CI: 83.8–100.0) at 6 months and 77.7% (95%CI: 63.4–95.2%) at 24 months (Figure 5) with primary assisted patency of 100% at 6 and 12 months and up to 66.7% at 24 months (Figure 6).

## 4. Discussion

Endovascular repair of TAAs or juxta-renal aneurysms has lower peri-operative morbidity and mortality compared with open repair [15].

Open repair requires suprarenal or coeliac cross-clamping, which would be unsuitable for the high proportion of our patients who have multiple comorbidities, rendering them ASA II [16,17,18,19,20].

A large proportion in our series comprises males with atherosclerotic risk factors such as hypertension at 83% and smoking at 70%, as well as previous aortic interventions such as TEVAR for the proximal aorta or even a prior open infrarenal AAA repair and coronary artery graft bypass (CABG). In addition, open repair renders an increased risk of renal impairment, with an estimated 3% of patients requiring hemodialysis. Other well-known complications include ischaemic stroke, cardiac complications and death, with a meta-analysis of 22 studies demonstrating a 30-day mortality rate of 4.4% for open repair of juxtarenal AAA [21].

Fenestrated or multi-branch endovascular aortic repair (FEVAR) is a well-established, safe and durable treatment option for TAAs with excellent clinical results in both emergent and elective settings [6,10,11,22,23,24]. These devices may be “off-the-shelf” or customised.

While “off-the-shelf devices” with standard multi-branches (4 branches) are anatomically suitable in 88% of patients and more readily available in emergencies, they are limited when the need for additional branches, retrograde-direction branches, or aortic diameters outside the standard size range arises [6,7,8,9,25].

Some studies have also shown concerns regarding their long-term branch stability, the tendency to kink in the setting of misaligned fenestration/branches and bridging stents, henceforth causing stenosis of target vessels with higher secondary reintervention rates [18,25,26].

Off-the-shelf devices preclude the incorporation of ARA and may result in additional aortic coverage with sometimes unnecessary inclusion of the visceral vessels [27]. Other techniques used for branch artery preservation include the chimney technique, which we use in emergent, time-sensitive settings and occasionally as a form of bail-out. [6,28]

Alteratively, physician-modified endograft created by modification of conventional available endografts have been described to overcome the limits of custom-made and off-the-shelf fenestrated devices with good results. However, they require much more experience and technical ability, and their long-term durability is still under-studied [6,29,30].

It is well known that the production of custom-made devices is costly, may take up to 16 weeks with potential lethal delays and hence be more suited for the elective setting [31,32].

Notably, few studies on the utility of custom-made devices have been reported in Southeast Asian or Asian regions where the vast majority of vascular patients have significant chronic limb-threatening ischemia, ischemic rather than aneurysmal disease [33,34].

The results of this study report the early clinical experience with the use of fenestrated COOK CMD at a single tertiary centre in Singapore from 2012 to 2022. More recently, we have also performed these cases with the ARTIVION endograft and used inner branch devices. However, the aim of this study is to look at these cases within at least a 2-year follow-up period. This study has catered for a mean follow-up of 32.4 months (range 1–108 months), which is comparable or longer and more recent than several of the studies on the use of CMDs for TAAs [6,10,11,12].

All stent grafts were successfully deployed without conversion to open or explant. Catheterisation and stenting of branches were successful in 94% of the cases, which is comparable to other series [6,10,11,12]. The majority of the fenestrations that could not be cannulated were the coeliac ones, and fortunately, most did not result in a significant proximal endoleak, and 2 of them were salvaged with a chimney technique. This could partly be due to the fact that the majority of these were Crawford IV aneurysms.

### 4.1. Branch Catheterization

Catheterisation was unsuccessful in only one renal artery, and no significant early renal impairment or need for dialysis was recorded.

ARA has been reported to be present unilaterally in 25% of the population and bilaterally in 10% [27,35]. In our series, only 3/29 (10%) of our cases had designs for ARA, admittedly providing us with little experience to describe. Coverage of ARA may lead to renal infarction and potentially long-term renal insufficiency, and preservation of ARAs with a diameter ≥ 4 mm or when they supplied one-quarter of the renal parenchyma has been recommended [28,29].

The use of the APTUS steerable sheath has been helpful in cases with prior proximal interventions, even in the presence of unfavourable angles, allowing some cases to be performed purely retrograde without upper limb access altogether.

In our series, we started to use the steerable sheath in the more recent cases from 2020 to 2022. It has also been helpful in re-interventions. Likewise, Kapahke S et al. demonstrated that with the use of a steerable sheath, despite having up to 67% of target vessels identified as challenging, a retrograde transfemoral approach was safe and feasible [36].

### 4.2. Peri-Procedure Complications

Our peri-procedural complication rates are broadly comparable with the available literature, with no significant haemorrhage, aortic perforation, need for graft explant, or recorded on-table mortality [6,10,11,12].

### 4.3. Access-Related Complications

Access complications formed the majority of these early complications, and they prolong hospital stays due to delayed mobility. These could be related to the atherosclerotic calcified nature of these access vessels, with a significant proportion of them having previous aortic or other endovascular interventions. In addition, it is well known that Asian blood vessels are smaller in diameter and potentially more tortuous [37]. Many of these complications also occurred in our earlier cases.

Fortunately, these were managed with open repair in the form of open embolectomy, bovine pericardial patch repair and rarely, the use of an interposition graft or fem-fem crossover bypass. Severely calcified arteries are at high risk of suture-mediated closure device failure, and perhaps the new edition may improve percutaneous closures of such groins [38]. Increased familiarity with closure devices and early identification of potentially difficult access groins have also helped minimise such complications.

Percutaneous brachial access complications likewise proved fairly common, and as such, many operators have elected to perform open exposure of the brachial artery for sheath insertion under direct vision with subsequent closure so as to prevent both pseudoaneurysm or vessel thrombosis similar to the study published by Nasr B et al. [39].

### 4.4. Spinal Drain and Ischemia

In our early practice, the spinal drain was prophylactically inserted and safely removed without major complications. Three patients had post-operative concerns of paraplegia, but these were transient and resolved, fortunately, when the spinal drain was unclamped. We note that this is not common practice in several other series [40], and moving on, we may review the routine need for this, albeit being a relatively low-risk procedure in itself. The overall rate of complications associated with lumbar CSF drainage is low at 6.5%, including bleeding, CSF leak and meningitis [41,42].

Joseph Brandreth et al. described that while the impact of cross-clamping is avoided in TEVAR, occlusion of branches of the aorta by the endograft, particularly the left subclavian artery but also other segmental branches, is the main cause of reduced blood flow to the spinal cord [43].

TEVAR at high risk of spinal complications includes those where extent of endograft coverage > 20 cm, stent graft placement covers origin of artery of Adamciewicz (T9-12), subclavian artery, internal iliac arteries, placement of 3 or more stents, Crawford classification I and II aneurysms, increased blood loss, previous aortic surgery, atheromatous aorta and patients with comorbidities such as chronic kidney disease, diabetes mellitus, hypertension, COPD, aortic dissection. Other considerations include female, anaemic and elderly patients or the development of perioperative hypotension as well. Spinal cord perfusion may also be compromised by embolism of atherosclerotic plaques from the thoracic aorta. The decision to insert a spinal drain preoperatively depends on a balance of risks and benefits, and the only absolute contraindication is patient refusal. Presentation of spinal cord ischemia may be immediate or delayed up to 48 h, and this delay is more common following TEVAR than open repair [35,44].

### 4.5. Follow-Up Outcomes and Complications

Follow-up data showed that no patients died from progression of aortic-related disease. The mean follow-up of our patients at the time of data collection was almost 3 years, with a range of 1 month to 9 years.

Sac enlargement in approximately one-third of these cases at follow-up is comparable to many studies, with the rest showing either sac regression or stability, which are predictors for good outcomes and less need for reintervention [45,46].

### 4.6. Reintervention—Occlusion and Endoleaks

One-third of the patients required reintervention over the course of follow-up, with 2/3 of these due to significant endoleak and sac progression, and the other third was due to renal or limb occlusion.

For complications related to the visceral branch or limb occlusion, the Angiojet Peripheral Thrombectomy system (Boston Scientific) proved most useful for endovascular mechanical thrombectomy. This is often followed by extension or relining of the involved fenestrations/branches (Figure 2).

Classical type II endoleaks are easily identified and treated, as in Figure 4, secondary to IMA. However, the instability of fenestration is more difficult to diagnose. The latter condition should be suspected in all sac reperfusions occurring around a fenestration, not in connection with an aortic side branch, even in the absence of frank stent disconnection [47]. A diagnostic angiogram with cannulation of the branches is most useful to help identify this branch-stent disconnect (Figure 3).

By employing this approach, we were able to report a 6-month intervention-free more than 90% of cases and up to 77% at 1 year with two-thirds interval-free after reintervention at 1 year.

### 4.7. Limitations

Unlike infra-renal abdominal aortic aneurysms, the current evidence comparing endovascular techniques for complex abdominal aortic aneurysms is limited to observational and population-based studies, with no randomised controlled trials [13]. These studies are heterogeneous in nature and consist of a mixture of endovascular techniques, including off-the-shelf, PMEG and chimney techniques.

Our retrospective study focuses on CMDs and is limited by the small sample size, which renders it much more descriptive in nature. From other similar studies, it is known that patients with dissection, previous aortic surgery and the need for an intraprocedural embolisation branch are statistically associated with the need for re-intervention [12].

Other non-Asian series by Halon et al. [10] and Katsargyris et al. [11] were retrospective non-Asian series comprising the COOK CMDs demonstrated similar follow-up intervals, perioperative complications, reintervention and mortality.

Haulon et al.’s series combined both retrospective and prospectively collected data from 1073 and 186 patients, respectively. All underwent FEVAR with the COOK CMD. Technical success was over 95%, with a mean follow-up of 3.2 (retrospective data) and 1.7 (prospective data) years. 30-day mortality was 5%, with a secondary reintervention rate for endoleaks at 15% and a 34.3% adverse event rate in the retrospective database. Endoleak rates at 2 years were similar, with Type II being the majority at 15%, type III at 4.9%, and type 1 distal at 6.5%

Katsargyris et al. presented 349 patients with the same device and a mean follow-up of 49 months. Technical success was achieved in 98% of cases, with 47 reinterventions performed in 38 patients, representing a rate of approximately 10%.

The cumulated experience and collaboration between vascular surgeons and interventional radiologists at our institution have allowed for technical success in these cases and for patients to benefit from endovascular rather than open repair of such challenging aneurysms. Even the decision to choose a custom or fenestrated device over persisting with standard EVAR and risking a type 1A endoleak can be better made by an experienced team [6,47]. Accurate pre-procedural planning is crucial, and over the years, we have developed fewer access-related complications, reduced operative times, and contrasted use and confidence with cannulation.

## 5. Conclusions

In conclusion, our study reports the first mid-long-term evolving real-world result of the use of CMD for TAA in a tertiary Singaporean centre with reasonable safety and feasibility. Re-intervention at 30% suggests that disease and procedures remain challenging, emphasising the need to assimilate lessons and experience at high-volume centres.

## Figures and Tables

**Table 1:** Demographics and Clinical Characteristics (* significance at *p* < 0.05).

Population Characteristics	Overall (N = 29)	No Reintervention (N = 20)	Reintervention (N = 9)	*p*-Value
Gender, Male	26 (89.7)	18 (90.0)	8 (88.9)	0.928
Mean Age, years (SD)	66.7 ± 11.6	69.2 ± 8.9	61.2 ± 15.2	0.15
**Risk factors**				
Hypertension	24 (82.8)	17 (85.0)	7 (77.8)	0.634
Diabetes	9 (31.0)	6 (30.0)	3 (33.3)	0.858
Dyslipidemia	21 (72.4)	16 (80.0)	5 (55.6)	0.173
Renal Insufficiency	10 (34.5)	7 (35.0)	3 (33.3)	0.930
Ex/Smoker	20 (69.0)	14 (70.0)	6 (66.7)	0.858
**Aortic/Cardiac Interventions**				
Elephant trunk/Great vessel transposition/Aortic–bifem/fem-fem bypass	0 (0.0)	0 (0.0)	0 (0.0)	-
Aortic valve surgery	2 (6.9)	0 (0.0)	2 (22.2)	0.029 *
TEVAR	2 (6.9)	1 (5.0)	1 (11.1)	0.548
EVAR	0 (0.0)	0 (0.0)	0 (0.0)	-
Open AAA repair	5 (17.2)	1 (5.0)	4 (44.4)	0.009 *
Open Ascending aorta repair	2 (6.9)	0 (0.0)	2 (22.2)	0.029 *
CABG	7 (24.1)	5 (25.0)	2 (22.2)	0.872
**Crawford**				0.383
4	19 (65.5)	12 (60.0)	7 (77.8)	
3	8 (27.6)	7 (35.0)	1 (11.1)	
2	0 (0.0)	0 (0.0)	0 (0.0)	
1	2 (6.9)	1 (5.0)	1 (11.1)	
Mean aneurysmal diameter (cm)	5.5 ± 1.0	5.4 ± 1.1	5.8 ± 0.5	0.193
Iliac Aneurysm > 3 cm	3 (10.3)	1 (5.0)	2 (22.2)	0.159

**Table 2:** Operative and Technical Details.

Technical Details	
Technical successful branch catheterisation	N = 109/116 (94%)
**Vessels**	
Coeliac Axis	21/27
SMA	29/29
Renal arteries	
Right	29/29
Left	26/27
accessory	3/3
Antegrade (from brachial) vs. retrograde	79 vs. 37
Use of steerable sheath for cannulation	Used in 3 cases, total 9 branches
Brachial access	22/29
Percutaneous	17 (3 patients bilateral brachial)
Upfront Open cut down for brachial access	5
Mean Operative times, hours (±SD)	5.5 ± 2.0
Mean Contrast Volume, mL (±SD)	340 ± 93
**Associated Surgical Interventions**	
Fem-Fem bypass	1
Femoral endarterectomy and patch plasty	6
**Intra-Procedural Embolization**	7/29
Internal Iliac Arteries	2
IMA	5
Accessory Renal Arteries	1

**Table 3:** Complications, Follow-up and Re-interventions.

Complications, Follow-Up and Re-Interventions	
Groin complication needing Surgical repair	4
Brachial complications needing surgical repair	4
Perioperative Morbidity	4, 2 CVA, 2 Myocardial infarction
Branch occlusions	All > 1 year incidental on surveillance 4 renal—3 patients, 1 bilateral underwent thrombolysis, 2 unilateral—treated conservatively
Aortic limb occlusion	2 iliac limbs, required thrombolysis and relining
Endoleak	12/29
Type 3	3
Type 2	6
Type 1	3
Late Mortality	4, non-aneurysmal related 3 malignancies 1 pneumonia
No endoleak within follow up period	17/29
Stable sac size reported	10
Sac size reduction reported	7

## Data Availability

The datasets presented in this article are not readily available because of the Personal Data Protection Act.
